# Irradiation-Induced Cardiac Connexin-43 and miR-21 Responses Are Hampered by Treatment with Atorvastatin and Aspirin

**DOI:** 10.3390/ijms19041128

**Published:** 2018-04-10

**Authors:** Csilla Viczenczova, Branislav Kura, Tamara Egan Benova, Chang Yin, Rakesh C. Kukreja, Jan Slezak, Narcis Tribulova, Barbara Szeiffova Bacova

**Affiliations:** 1 Institute for Heart Research, Center of Experimental Medicine, Slovak Academy of Sciences, Bratislava 841 04, Slovak Republic; viczencz.csilla@gmail.com (C.V.); branislav.kura@savba.sk (B.K.); tamara.benova@savba.sk (T.E.B.); jan.slezak@savba.sk (J.S.); narcisa.tribulova@savba.sk (N.T.); 2 Division of Cardiology, Medical College of Virginia, Virginia Commonwealth University, Richmond, VA 23298, USA; rakesh@vcu.edu (C.Y.); rakesh.kukreja@vcuhealth.org (R.C.K.)

**Keywords:** irradiation, heart, connexin-43, miR-1, miR-21, atorvastatin, aspirin

## Abstract

Radiation of the chest during cancer therapy is deleterious to the heart, mostly due to oxidative stress and inflammation related injury. A single sub-lethal dose of irradiation has been shown to result in compensatory up-regulation of the myocardial connexin-43 (Cx43), activation of the protein kinase C (PKC) signaling along with the decline of microRNA (miR)-1 and an increase of miR-21 levels in the left ventricle (LV). We investigated whether drugs with antioxidant, anti-inflammatory or vasodilating properties, such as aspirin, atorvastatin, and sildenafil, may affect myocardial response in the LV and right ventricle (RV) following chest irradiation. Adult, male Wistar rats were subjected to a single sub-lethal dose of chest radiation at 25 Gy and treated with aspirin (3 mg/day), atorvastatin (0.25 mg/day), and sildenafil (0.3 mg/day) for six weeks. Cx43, PKCε and PKCδ proteins expression and levels of miR-1 as well as miR-21 were determined in the LV and RV. Results showed that the suppression of miR-1 was associated with an increase of total and phosphorylated forms of Cx43 as well as PKCε expression in the LV while having no effect in the RV post-irradiation as compared to the non-irradiated rats. Treatment with aspirin and atorvastatin prevented an increase in the expression of Cx43 and PKCε without change in the miR-1 levels. Furthermore, treatment with aspirin, atorvastatin, and sildenafil completely prevented an increase of miR-21 in the LV while having partial effect in the RV post irradiation. The increase in pro-apoptotic PKCδ was not affected by any of the used treatment. In conclusion, irradiation and drug-induced changes were less pronounced in the RV as compared to the LV. Treatment with aspirin and atorvastatin interfered with irradiation-induced compensatory changes in myocardial Cx43 protein and miR-21 by preventing their elevation, possibly via amelioration of oxidative stress and inflammation.

## 1. Introduction

Cardiovascular injury due to radiation is the most common cause of adverse events among cancer survivors [1,2]. Key factors responsible for the establishment of cardiovascular injury, i.e., oxidative stress, inflammation, and epigenetic modifications, have been linked to potential treatments and been recently described [1,2]. Ionizing radiation induces oxidative stress and causes changes in the expression of several microRNAs (miRNA)s, including miR-1 and miR-21. An increase of miR-21 is involved in myocardial hypertrophy [3,4] and fibrosis [5]. An increase in miR-21 has also been associated with the up-regulation of the protein kinase C (PKC) δ [6], which is also implicated in tissue remodeling. Fibrosis and necrosis were reduced by the treatment with a free radical-scavenging component such as melatonin [7]. However, molecular mechanisms of the irradiation induced injury are unknown and there is currently a lack of treatment strategies.

Cardiac connexin-43 (Cx43) channels are essential for coordinated heart function because they ensure electrical coupling and direct intercellular communication. We and others [6,8] have shown that a single sub-lethal dose of irradiation results in up-regulation of Cx43, which has been associated with the protection of the heart against malignant arrhythmias [9] and infarction [10]. An increase in myocardial Cx43 expression and its active phosphorylated forms has been shown to be associated with the suppression of miR-1 (which regulates GJA1 gene transcription for Cx43) and the enhancement of PKCε (which phosphorylates Cx43) [6]. It appears that these early post-irradiation related myocardial alterations, including up-regulation of Cx43, are most likely compensatory responses of the heart to maintain its normal function [10,11,12]. Since irradiation induces inflammation and oxidative stress [12], we hypothesized that compounds exerting anti-inflammatory and antioxidant actions would interfere with irradiation-induced compensatory responses. To this context, we considered several drugs, which included acetylsalicylic acid (aspirin), a non-selective inhibitor of cyclooxygenase-1 and cyclooxygenase-2, which prevents formation of pro-inflammatory prostaglandins and thromboxanes. Antioxidant properties of acetylsalicylic acid are attributed to its ability of inhibiting lipid peroxidation and DNA damage [13]. Atorvastatin is widely used for treatment of human dislipidemia due to inhibition of the 3-hydroxy-3-methylglutaryl coenzyme A reductase. In addition, its pleiotropic effects are associated with anti-inflammatory and antioxidative actions [14] as well as promoting the availability of vascular nitric oxide [15]. Sildenafil, the inhibitor of phosphodiestherase 5, is used for its vasodilatation and activation of nitric oxide [16].

Our goal was to demonstrate whether treatment with the drugs targeting oxidative stress and inflammation might result in attenuation of irradiation induced myocardial compensatory responses previously reported [6,10]. In particular, we examined myocardial changes in Cx43, PKC, miR-1 and miR-21 in rats exposed to single chest irradiation.

## 2. Results

### 2.1. Main Characteristics of Experimental Rats

Comparing to non-irradiated rats, the body, heart, and left ventricular weight were significantly decreased in irradiated animals after six weeks. On the other hand, irradiation did not alter the right ventricular weight. Treatment with selected drugs also had no effect on these biometric parameters in any treated group, except for the heart weight in post-irradiated + sildenafil group. Data are summarized in the Table 1.

### 2.2. Protein Expression of Myocardial Cx43 in Control and Irradiated Wistar Rats

**Figure 1:**
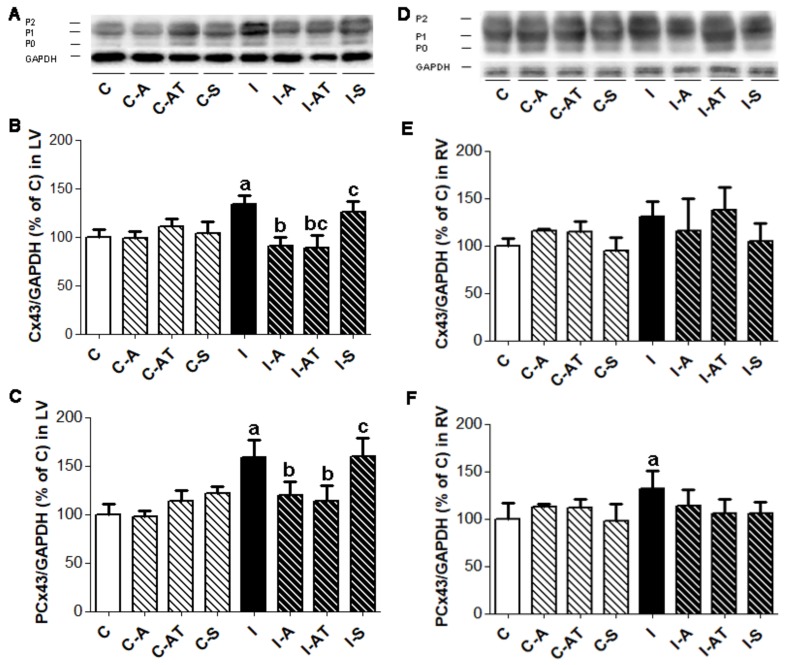
Representative immunoblots showing three forms of Cx43 (**A**,**D**) and densitometric quantification of total Cx43 expression (**B**,**E**), and its phosphorylated forms (**C**,**F**) normalized to GAPDH in the LV (left panel) and RV (right panel) of non-irradiated and post-irradiated Wistar rats with and without treatment. Abbreviations—P0: unphosphorylated form of Cx43; P1 and P2: phosphorylated forms of Cx43; GAPDH: housekeeper; C: control non-irradiated rats; C-A: control + aspirin; C-AT: control + atorvastatin; C-S: control + sildenafil; I: post-irradiated rats; I-A: post-irradiated + aspirin; I-AT: post-irradiated + atorvastatin; I-S: post-irradiated + sildenafil. Data are means ± SD of 6 hearts. ^a^ *p* < 0.05 vs. C (C vs. C-A, C-AT, C-S, I); **^b^** *p* < 0.05 vs. I (I vs. I-A, I-AT, I-S); ^c^ *p* < 0.05 treated C vs. treated I (C-A vs. I-A; C-AT vs. I-AT; C-S vs. I-S).

Western blot analysis showed that total Cx43 protein was increased in the LV (*p* < 0.05) and to a lesser extent in RV post-irradiation as compared to the non-irradiated group (Figure 1A,B,D,E). In parallel, the active phosphorylated forms of Cx43 were significantly increased in the LV and RV of irradiated rats versus the non-irradiated controls (Figure 1A,C,D,F). Treatment with aspirin and atorvastatin for six weeks (starting one day before irradiation) suppressed the elevation of the total as well as the phosphorylated forms of Cx43, significantly in the LV (Figure 1A–C) while having no effect in RV (Figure 1D–F) of post-irradiated animals. The administration of sildenafil had no significant effect on the irradiation-induced increase in myocardial Cx43, i.e., either on total levels or its phosphorylated forms after six weeks (Figure 1).

### 2.3. Expression of Protein Kinase Cε in Control and Irradiated Wistar Rats

**Figure 2:**
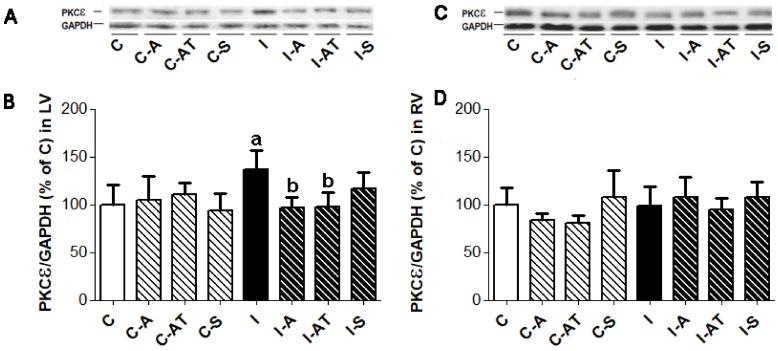
Representative immunoblots of PKCε expression (**A**,**C**) and its quantitative evaluation normalized to GAPDH in the LV ((**B**), left panel) and RV ((**D**), right panel) of non-irradiated and post-irradiated Wistar rats with and without treatment. Abbreviations—PKCε: protein kinase C epsilon; GAPDH: housekeeping protein; C: control non-irradiated rats; C-A: control + aspirin; C-AT: control + atorvastatin; C-S: control + sildenafil; I: post-irradiated rats; I-A: post-irradiated + aspirin; I-AT: post-irradiated + atorvastatin; I-S: post-irradiated + sildenafil. Data are means ± SD of 6 hearts. ^a^ *p* < 0.05 vs. C (C vs. C-A, C-AT, C-S, I); ^b^ *p* < 0.05 vs. I (I vs. I-A, I-AT, I-S).

PKCε was significantly increased in the LV (Figure 2A,B) but not in the RV (Figure 2C,D) of rats following irradiation when compared to the non-irradiated controls. Six weeks of treatment with aspirin and atorvastatin following irradiation normalized PKCε in the LV (Figure 2A,B) while having no effect in the RV (Figure 2C,D). Treatment with sildenafil had no effect on the PKCε expression of the irradiated rats. There was no change in the expression of PKCε in non-irradiated rats following treatment with drugs (Figure 2).

### 2.4. Expression of Protein Kinase C δ in Control and Irradiated Wistar Rats

**Figure 3:**
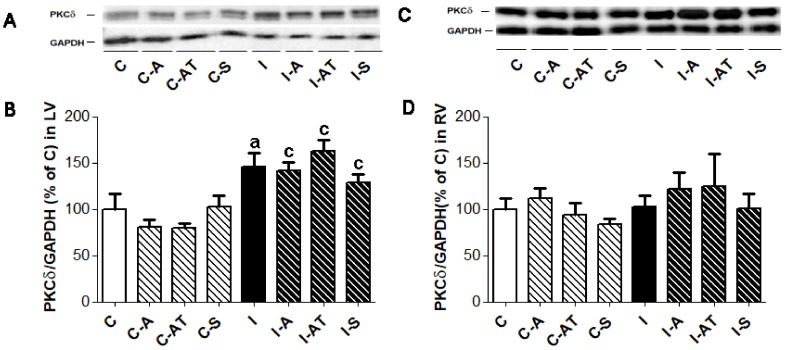
Representative immunoblots of PKCδ expression (**A**,**C**) and quantitative evaluation normalized to GAPDH in the LV ((**B**), left panel) and RV ((**D**), right panel) of non-irradiated and post-irradiated Wistar rats. Abbreviations—PKCδ: protein kinase C delta; GAPDH: housekeeping protein; C: control non-irradiated rats; C-A: control + aspirin; C-AT: control + atorvastatin; C-S: control + sildenafil; I: post-irradiated rats; I-A: post-irradiated + aspirin; I-AT: post-irradiated + atorvastatin; I-S: post-irradiated + sildenafil. Data are means ± SD of 6 hearts. ^a^ *p* < 0.05 vs. C (C vs. C-A, C-AT, C-S, I); ^c^ *p* < 0.05 treated C vs. treated I (C-A vs. I-A; C-AT vs. I-AT; C-S vs. I-S).

Similar to PKCε, the myocardial expression of PKCδ (Figure 3) was increased in response to irradiation. The increase was significant in the LV (Figure 3A,B) while no change was observed in the RV (Figure 3C,D) when compared to the to non-irradiated controls. Treatment with drugs had no effect on the expression of PKCδ in the LV or RV in the irradiated and non-irradiated groups (Figure 3).

### 2.5. Myocardial Expression of miR-1 in Control and Irradiated Wistar Rats

**Figure 4:**
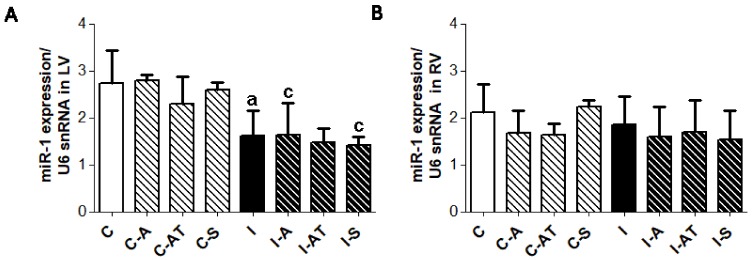
Myocardial expression of miR-1 in the LV ((**A**), left panel) and RV ((**B**), right panel) of non-irradiated and post-irradiated Wistar rats with and without treatment. Endogenous U6 small nuclear RNA (U6snRNA) was used to normalize miR-1. Abbreviations—C: control non-irradiated rats; C-A: control + aspirin; C-AT: control + atorvastatin; C-S: control + sildenafil; I: post-irradiated rats; I-A: post-irradiated + aspirin; I-AT: post-irradiated + atorvastatin; I-S: post-irradiated + sildenafil. Data are means ± SD of 5 hearts. ^a^ *p* < 0.05 vs. C (C vs. C-A, C-AT, C-S, I); ^c^ *p* < 0.05 treated C vs. treated I (C-A vs. I-A; C-AT vs. I-AT; C-S vs. I-S).

miR-1 level decreased following six weeks after irradiation (Figure 4), which was signficant in the LV (Figure 4A) but not in the RV (Figure 4B). Treatment with the drugs had no significant effect on miR-1 level in post-irradiated as well as in non-irradiated groups (Figure 4).

### 2.6. Expression of miR-21 in Control and Irradiated Wistar Rats

**Figure 5:**
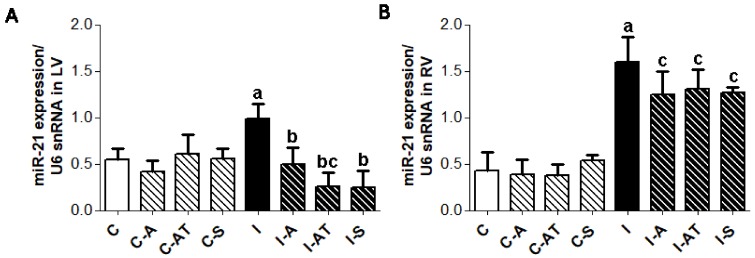
Myocardial expression of miR-21 in the LV (**A**) and RV (**B**) of non-irradiated and post-irradiated Wistar rats with and without treatment. Endogenous U6 small nuclear RNA (U6snRNA) was used to normalize miR-21. Abbreviations—C: control non-irradiated rats; C-A: control + aspirin; C-AT: control + atorvastatin; C-S: control + sildenafil; I: post-irradiated rats; I-A: post-irradiated + aspirin; I-AT: post-irradiated + atorvastatin; I-S: post-irradiated + sildenafil. Data are means ± SD of 5 hearts. ^a^ *p* < 0.05 vs. C (C vs. C-A, C-AT, C-S, I); ^b^ *p* < 0.05 vs. I (I vs. I-A, I-AT, I-S); ^c^ *p* < 0.05 treated C vs. treated I (C-A vs. I-A; C-AT vs. I-AT; C-S vs. I-S).

The expression of myocardial miR-21 was significantly increased in both the LV and RV of the post-irradiated rats compared to the non-irradiated controls (Figure 5). Six weeks of treatment with the selected drugs significantly suppressed miR-21 expression in the left (Figure 5A), although to a lesser extent in the RV (Figure 5B) following irradiation. There was no effect of drugs on miR-21 levels in the non-irradiated control groups (Figure 5).

## 3. Discussion

In the present study, we showed that exposure of rats to a single dose of chest irradiation at 25 Gy caused up-regulation in the expression of Cx43, PKCε, and PKCδ in the LV following six weeks. In parallel, miR-1, which is known to repress GJA1 for Cx43 [3,6] was decreased. While miR-21, which is involved in myocardial remodeling and apoptosis [3,6], was increased following irradiation. These results are in accordance with our previous findings [6,10] and in line with the reported enhancement of myocardial Cx43 protein and mRNA expression found in the rabbit heart in response to heavy ion radiation [8,9]. Importantly, these alterations were associated with protection of the heart against arrhythmia and infarction [8,10].

In the present study, we have also demonstrated that irradiation did not induce significant changes in the expression of total Cx43 in the RV, although there was a trend towards an increase. This may be in part due to the miR-1, which was not changed in the RV in contrast to its significant suppression associated with the enhancement of Cx43 in the LV. Furthermore, neither PKCε nor PKCδ expression was altered in the RV unlike its significant elevation in LV following irradiation. Whether such distinct responses to irradiation in the LV and RV have any relationship with functional, metabolic, structural, and other differences of the heart chambers [17,18] needs to be investigated. Chamber related differences in Cx43 and PKC expression in response to altered thyroid status have been previously suggested [19]. A higher metabolic rate and mechanical load of the LV seems to be more prone to oxidative stress and injury. Nevertheless, our results suggest that cardiac response to chest irradiation is associated with the up-regulation of myocardial Cx43 connected with the suppression of miR-1 and enhanced PKCε and PKCδ signaling in the LV but not in the RV. On the other hand, the expression of miR-21 was significantly increased in both the LV and RV following irradiation. It is possible that the anti-apoptotic [20] and proliferation promoting effects of miR-21 [5] may be pro-survival to cardiac tissue during the early period in response to radiation-induced injury.

Our results also show that treatment of the irradiated rats with aspirin and atorvastatin prevented up-regulation of Cx43, i.e., its total and phosphorylated forms in the LV. Interestingly, none of the drugs reduced the level of miR-1 in the LV following irradiation. These data suggest that apart from the miR-1, other post-translational factors may modulate Cx43 levels in response to irradiation. Neither atorvastatin nor aspirin had any effect on the basal myocardial expression of Cx43 in the non-irradiated control rats.

The question arises, how aspirin or atorvastatin may affect changes in myocardial Cx43 protein levels induced by single chest irradiation? Considering that both compounds exert protection from oxidative stress and inflammation—the important culprits in irradiation-induced cardiac injury [1,2]—it is reasonable to speculate that these drugs may suppress these processes. If so, then cardiac stress would be attenuated and a subsequent compensatory response (most likely mediated by free radicals and pro-inflammatory molecules signaling) will be reduced. Indeed, the administration of antioxidants and/or scavengers of free radicals, such as hesperidin [21], melatonin [7] or selenium [22] prior to irradiation attenuated oxidative stress, inflammation, and cardiomyocyte necrosis. The potential use of statins as radioprotective agents has been recently reviewed, where signaling pathways targeting pro-inflammatory NF-κB might be implicated [1]. In support of this concept, it should be noted that unlike aspirin and atorvastatin, treatment with sildenafil (dominant vasculo-protective drug) did not affect the myocardial Cx43 levels in irradiated rats. Moreover, aspirin and atorvastatin but not sildenafil reduced expression of TNF-α in irradiated rats in our model, as reported previously [23]. 

In general, oxidative stress and/or inflammation contribute to the impairment of intercellular communication due to the acceleration of Cx43 and Cx43 interacting proteins that are in degradation and/or dysfunction [24]. Consequently, down-regulation of Cx43 due to chronic redox disorders and subclinical inflammation often accompanies cardiovascular disease [25,26]. On the other hand, acute heart injury (e.g., intermittent ischemia) triggers endogenous pro-survival molecular pathways, including up-regulation of Cx43, to protect heart function [27]. It appears that the heart may respond to stressors by compensatory/adaptive (such as up-regulation of Cx43) and by maladaptive/decompensatory changes (down-regulation of Cx43). Accordingly, enhanced Cx43 level observed in the compensatory hypertrophy was induced by pressure overload, while reduced Cx43 occurred in the decompensatory state [25].

Our results also show that the treatment of irradiated rats with aspirin and atorvastatin normalized the myocardial expression of PKCε, which is associated with the expression of phosphorylated forms of Cx43. These results suggest that oxidative stress and inflammation may modulate myocardial PKCε, signaling and its cardioprotective role as shown in various conditions [28]. Interestingly, treatment did not affect elevated myocardial PKCδ expression, i.e., its pro-hypertrophic signaling in irradiated rat heart. However, the expression of pro-fibrotic miR-21 was significantly decreased in the left heart ventricle by all tested drugs. Of note, miR-21 has been shown up-regulated in human fibroblast cells due to various stress-inducing conditions, including radiation [3,29]. Thus, more attention should be paid to elucidate the possible anti-fibrotic effects of atorvastatin, aspirin, and perhaps sildenafil. Importantly, microRNA-21 has been reported as a novel promising target in cancer radiation therapy [20] and atorvastatin was shown to suppress expansive remodeling by inhibition of macrophage infiltration [30].

Taken together, we assume that protection of the heart from oxidative stress and inflammation by appropriate drugs, including aspirin and atorvastatin or nonpharmacological compounds like melatonin [7], omega-PUFA [31] may counteract compensatory responses and attenuate adverse consequences of chest irradiation.

## 4. Materials and Methods 

Animal experiments were approved by the Animal Research and Care Committee of the Institute for Heart Research, Slovak Academy of Sciences—Project 1873/11-221/3, approved on 30 September 2011 and in accordance with the rules issued by the State Veterinary Administration of the Slovak Republic, legislation No 289/2003. Rats were maintained on a 12:12 h’s light/dark cycle with access to standard pellet and water *ad libitum*.

### 4.1. Experimental Model of Irradiation Induced Cardiac Injury

Three-month-old male Wistar rats were randomly divided into irradiated (*n* = 24) and non-irradiated (*n* = 24) groups. Rats were anesthetized with Narketan (115 mg/kg body weight) followed by myorelaxant Xylan (1 mg/kg body weight) and exposed to a single dose of 25 Gy of ionizing radiation given locally on mediastinum at the area of the heart using the electron linear accelerator UELR 5-1S (Producer NIIEFA St. Petersburg, RF, Russia), as described previously [6]. Control animals were shielded with lead plates. Non-irradiated controls (C) and irradiated (I) rats were treated one day before, the day of irradiation, and for the next six weeks with aspirin (A; 3 mg/day), atorvastatin (AT; 0.25 mg/day) or sildenafil (S; 0.3 mg/day) via a gastric tube. Doses of the drugs were calculated from the maximal therapeutic dose for humans in relation to the rat body weight.

At the end of the experiment, hearts were excised from the anesthetized animals (thiopental, 65 mg/kg body weight) followed by the registration of the whole heart as well as left (LV) and right ventricle (RV) weight. Frozen tissues from the heart ventricles were stored at −80 °C in freezer box and used for the analysis of Cx43, PKCε and PKCδ expression by the Western blot method and for miR-1 and miR-21 expression analysis by qRT-PCR.

### 4.2. Determination of Myocardial Cx43, PKCε and PKCδ Protein Expression

The western blot analysis was performed as previously described [6]. Briefly, the ventricular samples (*n* = 6 per group) were powdered and solubilized in SB20 (20% SDS, 10 mmol/L EDTA, 0.1 mol/L tris(hydroxymethyl) aminomethane (TRIS), pH 6.8 by sonicator UP 100H (Hielscher, Teltow, Germany). Total proteins (10–30 μg) from each sample were separated in 10% sodium dodecyl sulfate polyacrylamide gels, transferred onto a nitrocellulose membrane, and blocked with 5% non-fat dry milk in Tris-buffered saline. For the determination of Cx43, the membrane was incubated with a primary rabbit polyclonal antibody (diluted 1:4000; Anti-Connexin 43 C 6219; Sigma-Aldrich, St. Louis, MO, USA). For PKCε and PKCδ determination, the nitrocellulose membrane was incubated with primary rabbit polyclonal antibodies (PKCε Antibody, C-15: sc-214, Santa Cruz Biotechnology, Inc., PKCδ Antibody, C-17: sc-213, Santa Cruz Biotechnology, Inc., Santa Cruz, CA, USA) diluted 1:1000, overnight at 4 °C, followed by further incubation for 1 h at room temperature with a secondary donkey antibody (peroxidase-labeled anti-rabbit, 1:2000, Amersham Biosciences, Piscataway, NJ, USA). After ECL visualization, the densitometric analysis by Carestream Molecular Imaging Software (version 5.0, Carestream Health, New Haven, CT, USA.) was done using a KODAK In-Vivo Multispectral System FX. The measured values were normalized to the expression of GAPDH (glyceraldehyde-3-phosphate dehydrogenase) serving as a control [6].

### 4.3. Estimation of Myocardial miR-1 and miR-21 Levels

miR reverse transcription and TaqMan-based qRT-PCR analysis were performed as previously reported [6]. Total RNA including small RNA was isolated from the frozen ventricular tissue of the non-irradiated (*n* = 5 per group) and irradiated (*n* = 5 per group) Wistar rats using a miRNA mini kit according to the manufacturer’s protocol (QIAGEN Sciences, Germantown, MD, USA). The concentration and purity of the isolated RNA was checked using a Nanodrop ND-1000 spectrophotometer (Agilent Technologies, Santa Clara, CA, USA). Briefly, 10 ng of total RNA were subjected for reverse transcription reaction with miRNA specific RT primers using microRNA reverse transcription kit (Applied Biosystems, Foster City, CA, USA) in accordance to the manufacturer’s instructions. Real time PCR was performed using a Roche Light cycler 480 II (Roche Applied Science, Indianapolis, IN, USA). TaqMan miRNA assay probe (Applied Biosystems, Foster City, CA, USA) was applied to determine the expression level of miR-1 and miR-21. Endogenous U6 small nuclear RNA was used to normalize RNA content. Reverse transcription was performed using stem loop specific microRT primers under the following conditions: 16 °C for 30 min, 42 °C for 30 min, and 85 °C for 5 min. The obtained cDNA was diluted in 1:3 ratios and subjected to real-time PCR using a TaqMan amplicon specific assay probe under the following PCR cycle conditions: 95 °C for 10 min, 95 °C for 15 s, and 60 °C for 60 s [6].

### 4.4. Statistical Analysis

Data are expressed as means ± SD. One way two-tailed ANOVA and Tukey post hoc tests were used for statistical analysis. *p* < 0.05 was considered as statistically significant.

## 5. Conclusions

In conclusion, we have demonstrated that irradiation related changes are less pronounced in the RV as compared to the LV. Our results also suggest that treatment with aspirin and atorvastatin attenuate irradiation-induced up-regulation of myocardial Cx43 and PKCε signaling as well as miR-21 expression. Whether treatment related prevention or attenuation of irradiation-induced myocardial alterations at the early responsive period might help to prevent adverse late effects needs further investigations.

## Figures and Tables

**Table 1:** Biometric parameters registered in control and irradiated Wistar rats.

GROUP	BW (g)	HW (g)	LVW (g)	RVW (g)
C	344.22 ± 28.40	0.89 ± 0.11	0.36 ± 0.07	0.09 ± 0.02
C-A	382.61 ± 32.19	0.92 ± 0.09	0.39 ± 0.04	0.12 ± 0.01
C-AT	371.01 ± 20.45	0.87 ± 0.07	0.37 ± 0.04	0.10 ± 0.02
C-S	363.63 ± 23.93	0.90 ± 0.10	0.39 ± 0.04	0.11 ± 0.02
I	252.24 ± 14.31 ^a^	0.83 ± 0.05	0.25 ± 0.04 ^a^	0.10 ± 0.04
I-A	227.41 ± 26.47 ^c^	0.73 ± 0.09 ^c^	0.24 ± 0.02 ^c^	0.11 ± 0.03
I-AT	253.23 ± 31.82 ^c^	0.77 ± 0.08	0.27 ± 0.03 ^c^	0.13 ± 0.03
I-S	233.62 ± 14.50 ^c^	0.67 ± 0.05 ^b,c^	0.23 ± 0.02 ^c^	0.12 ± 0.03

BW—body weight; HW—heart weight; LVW—left ventricular weight; RVW—right ventricular weight; C—control non-irradiated; C-A—control + aspirin; C-AT—control + atorvastatin; C-S—control + sildenafil; I—irradiated rats; I-A—irradiated + aspirin; I-AT—irradiated + atorvastatin; I-S—irradiated + sildenafil. Results are the mean ± SD of 6 hearts. ^a^ *p* < 0.05 vs. C (C vs. C-A, C-AT, C-S, I); ^b^ *p* < 0.05 vs. I (I vs. I-A, I-AT, I-S); ^c^ *p* < 0.05 treated C vs. treated I (C-A vs. I-A; C-AT vs. I-AT; C-S vs. I-S).
